# Supplementary material for: An Automated Image Processing Module for Quality Evaluation of Milled Rice
Source: Foods. 2023 Mar 16;12(6):1273. doi: 10.3390/foods12061273 (PMC10048426; doi:10.3390/foods12061273)
Supplement: Supplementary file 1 [file foods-12-01273-s001.zip › foods-2244397-supplementary.pdf]

**Supplementary Table S1.** Market price and number of grains of eight types of rice varieties

| S. No. | Rice variety | Sample nomenclature | Market price (in INR Per kg) | Count of individual grain images |
|---|---|---|---|---|
| 1 | Traditional Basmati | BM | 78.0 | 295 |
| 2 | Eco Kolam | EK | 39.5 | 233 |
| 3 | HMT Kolam | HK | 45.5 | 363 |
| 4 | Kana Basmati | KB | 32.5 | 521 |
| 5 | Sona Masuri | SM | 30.5 | 402 |
| 6 | Tibar Basmati | TB | 61.5 | 431 |
| 7 | Tukda Basmati | TKB | 44.5 | 398 |
| 8 | Wada Kolam | WK | 48.5 | 438 |

**Supplementary Table S2.** Geometrical and morphological features of rice grains

| S. No. | Feature | Symbol | Mathematical Expression |
|---|---|---|---|
| **1** | Area | $A$ | - |
| **2** | Perimeter | $P$ | - |
| **3** | Area of bounding ellipse | $A_{ellipse}$ | $(\pi/4)L_{major}L_{minor}$ |
| **4** | Length of major axis of ellipse | $L_{major}$ | - |
| **5** | Length of minor axis of ellipse | $L_{minor}$ | - |
| **6** | Eccentricity of bounding ellipse | $e$ | - |
| **7** | Aspect ratio | $AR$ | $L_{major}/L_{minor}$ |
| **8** | Area of bounding rectangle | $A_{rectangle}$ | - |
| **9** | Area of convex hull | $A_{hull}$ | - |
| **10** | Extent | $E$ | $A_{rectangle}/A$ |
| **11** | Solidity | $S$ | $A_{hull}/A$ |
| **12** | Roundness | $R$ | $4\pi A/P^2$ |
| **13** | Equivalent diameter | $D_{eq}$ | $\sqrt{4A/\pi}$ |
| **14** | Compactness | $C$ | $D_{eq}/L_{major}$ |
| **15** | Shape factor 1 | $SF_1$ | $L_{major}/A$ |
| **16** | Shape factor 2 | $SF_2$ | $L_{minor}/A$ |
| **17** | Shape factor 3 | $SF_3$ | $A/\pi L_{major}^2$ |
| **18** | Shape factor 4 | $SF_4$ | $A/\pi L_{minor}L_{major}$ |

**Supplementary Table S3.** Summary of feature dataset

| Feature type | Symbol | Count |
|---|---|---|
| Geometrical and morphological | Listed in Table 2 | 18 |
| Textural | $CORR_i, CON_i, DIS_i, ASM_i, EN_i, HO_i$ for $i \in [0^{\circ}, 45^{\circ}, 90^{\circ}, 135^{\circ}]$ | 24 |
| Color | $R_i, G_i, B_i, H_i, S_i, V_i$ where $i \in [0,46]$ | 282 |

**Supplementary Table S4.** Number of actual versus the predicted grains in the validation samples

| Number of actual (A) and predicted (P) grains | | | | | | | | |
|---|---|---|---|---|---|---|---|---|
| | **Sample 1** | | **Sample 2** | | **Sample 3** | | **Sample 4** | |
| **Rice type** | **A** | **P** | **A** | **P** | **A** | **P** | **A** | **P** |
| **BM** | 50 | 41 | 0 | 7 | 0 | 0 | 50 | 39 |
| **EK** | 0 | 0 | 0 | 3 | 30 | 33 | 0 | 5 |
| **HK** | 0 | 2 | 0 | 3 | 0 | 4 | 0 | 3 |
| **KB** | 0 | 0 | 30 | 26 | 0 | 1 | 0 | 0 |
| **SM** | 0 | 1 | 0 | 5 | 0 | 3 | 0 | 9 |
| **TB** | 30 | 29 | 50 | 23 | 0 | 0 | 0 | 9 |
| **TKB** | 0 | 3 | 0 | 5 | 0 | 0 | 30 | 8 |
| **WK** | 0 | 0 | 0 | 0 | 50 | 38 | 0 | 2 |

**Supplementary Table S5.** Confusion matrix for the Random Forest classifier

| Predicted label \ True label | BM | EK | HK | KB | SM | TB | TKB | WK |
|---|---|---|---|---|---|---|---|---|
| BM | 56 | 0 | 0 | 0 | 0 | 3 | 0 | 0 |
| EK | 0 | 33 | 3 | 0 | 4 | 0 | 8 | 4 |
| HK | 0 | 5 | 35 | 0 | 3 | 0 | 5 | 14 |
| KB | 0 | 0 | 0 | 109 | 3 | 0 | 3 | 1 |
| SM | 0 | 1 | 0 | 6 | 70 | 0 | 6 | 0 |
| TB | 7 | 0 | 3 | 0 | 2 | 47 | 4 | 0 |
| TKB | 0 | 1 | 9 | 3 | 4 | 9 | 56 | 2 |
| WK | 0 | 2 | 9 | 2 | 3 | 0 | 1 | 62 |

BM, Basmati; EK, Eco Kolam; HK, HMT Kolam; KB, Kana Basmati; SM, Sona Masuri; TB, Tibar Basmati; TKB, Tukda Basmati; WK, Wada Kolam.

**Figure captions**

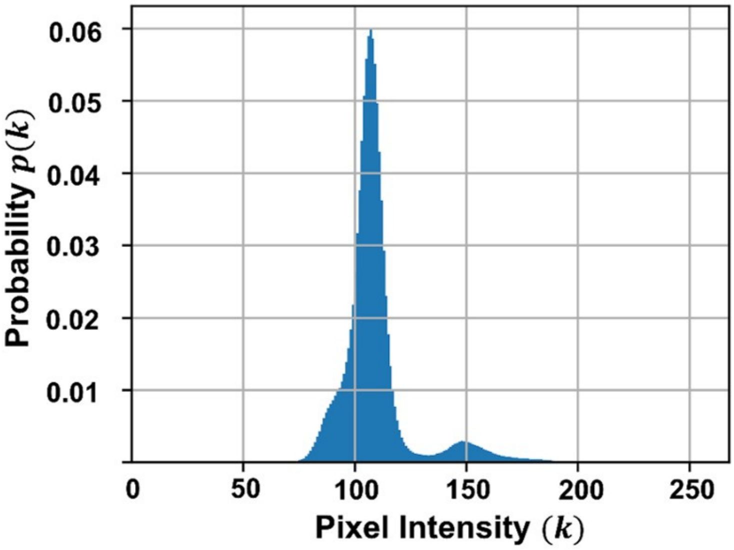


**Supplementary Figure S1.** The histogram for a representative gray scale image of rice grains.

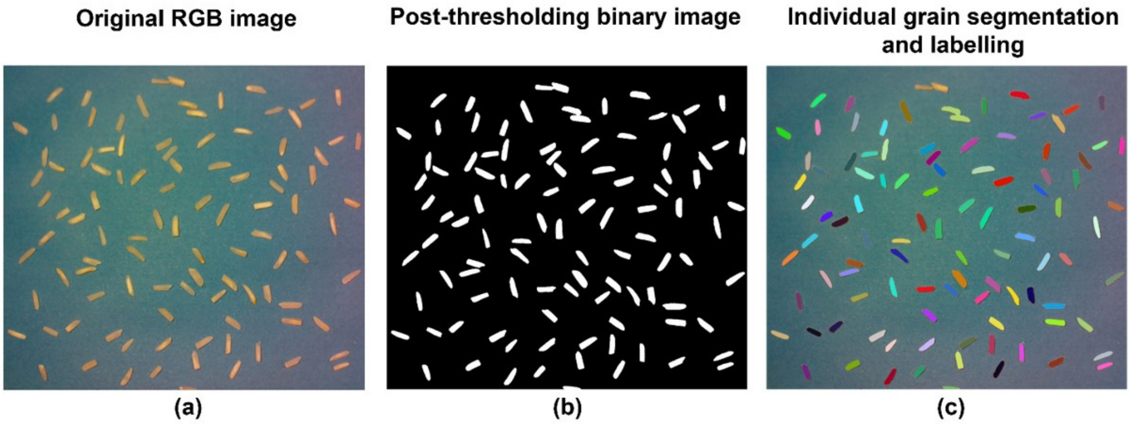


**Supplementary Figure S2.** Images of rice grains: (a) original RGB format, (b) gray scale format after global thresholding, and (c) individual labelled rice grains obtained using the Watershed algorithm.

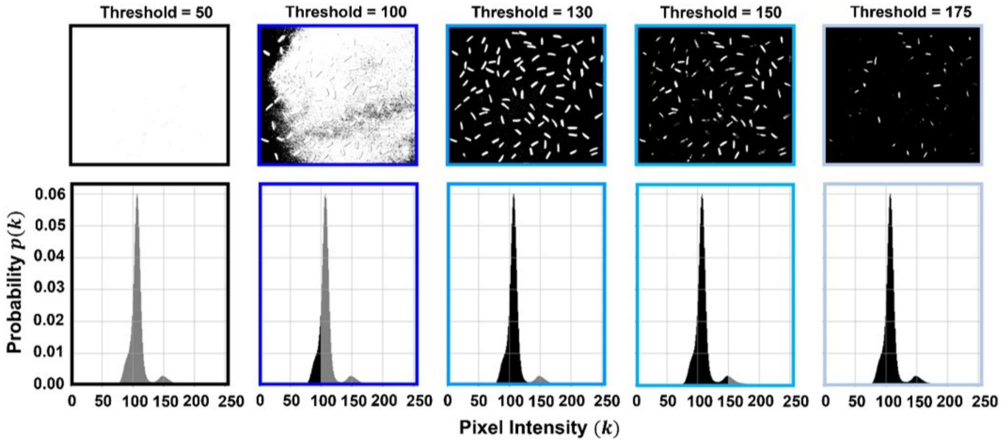


| Threshold = 50 | | 100 | 130 | 150 | 175 |
|---|---|---|---|---|---|
| $U_o$ | 0.00 | 0.21 | 0.92 | 0.96 | 0.99 |
| $U_1$ | 1.00 | 0.79 | 0.08 | 0.04 | 0.01 |
| $m_o$ | 47.22 | 93.17 | 104.91 | 106.39 | 108.28 |
| $m_1$ | 108.92 | 113.11 | 154.13 | 164.33 | 185.79 |
| $V_b^2$ | 0.43 | 66.10 | 180.81 | 139.46 | 48.19 |

**Supplementary Figure S3.** Estimation of global threshold value using Otsu's technique: (Top) Grayscale images of rice grains at different levels of thresholding. The corresponding histograms are shown below each image. (Bottom) The parameters used for obtaining the threshold values are tabulated.

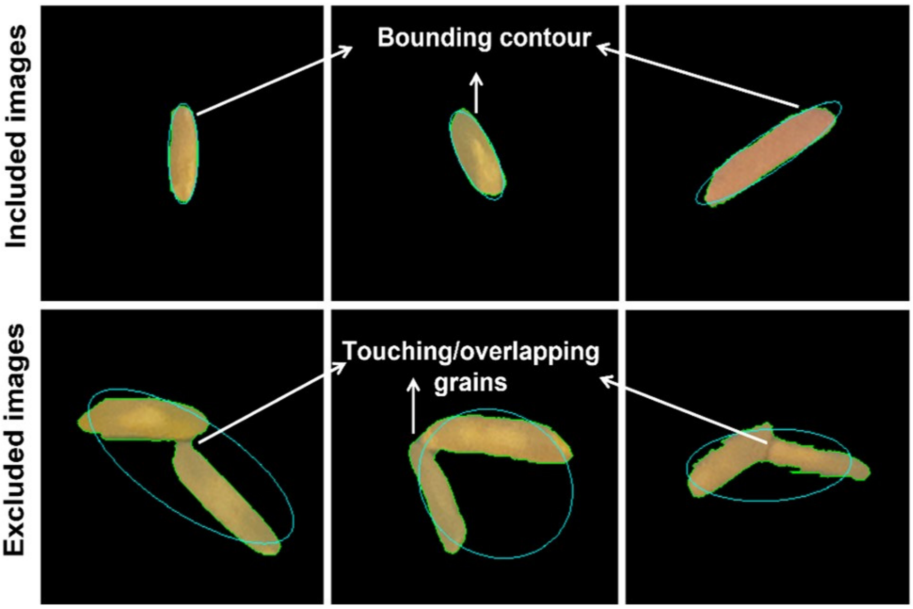


**Supplementary Figure S4.** Representative images of the segmented rice grains: (Top row) images of individual rice grains with bounding contours that were selected for image dataset. (Bottom row) images of rice grains that are overlapping/touching, and therefore, omitted from the dataset.

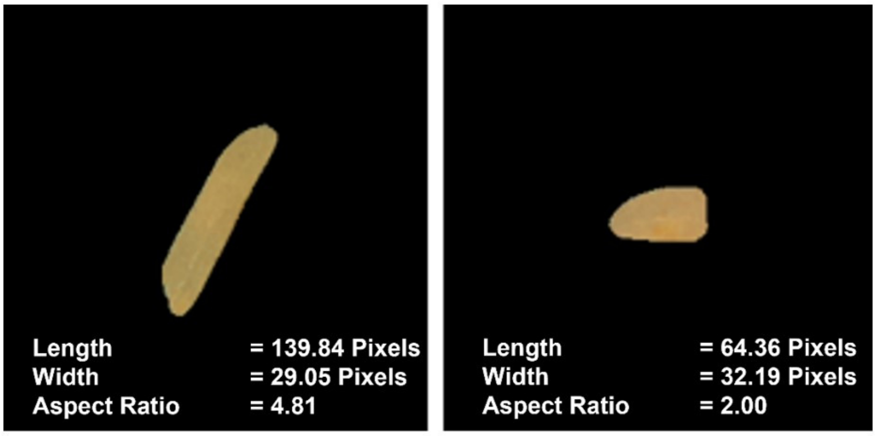


**Supplementary Figure S5.** Representative images showing selective geometrical features (length, width and aspect ratio) calculated for segmented rice grains.

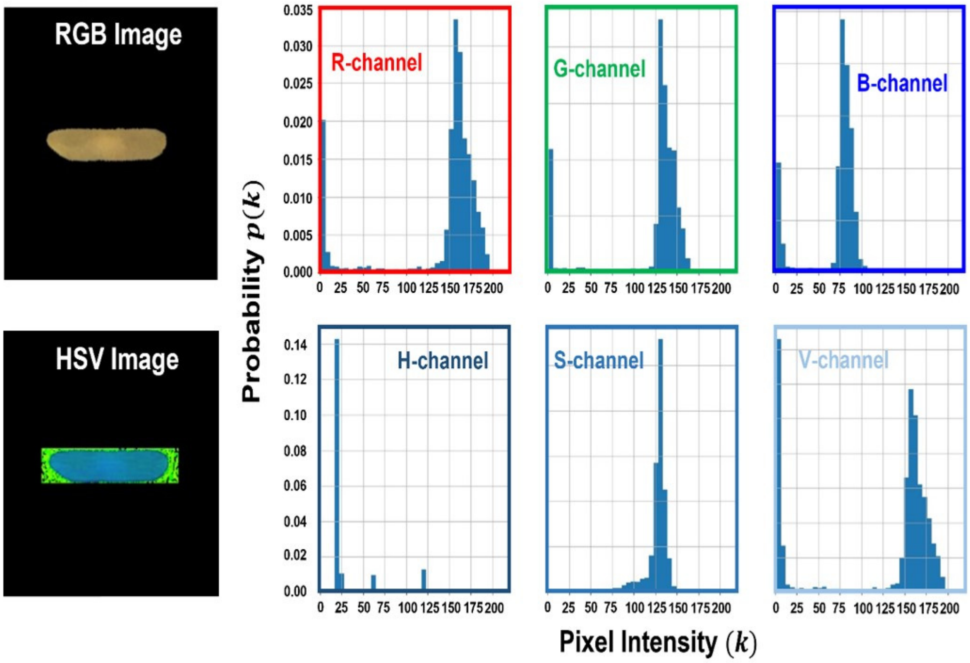


**Supplementary Figure S6.** (Left) RGB and HSV images of a segmented rice grain. (Right) histograms corresponding to the R, G, B channels (top row), and H, S, V channels (bottom row).

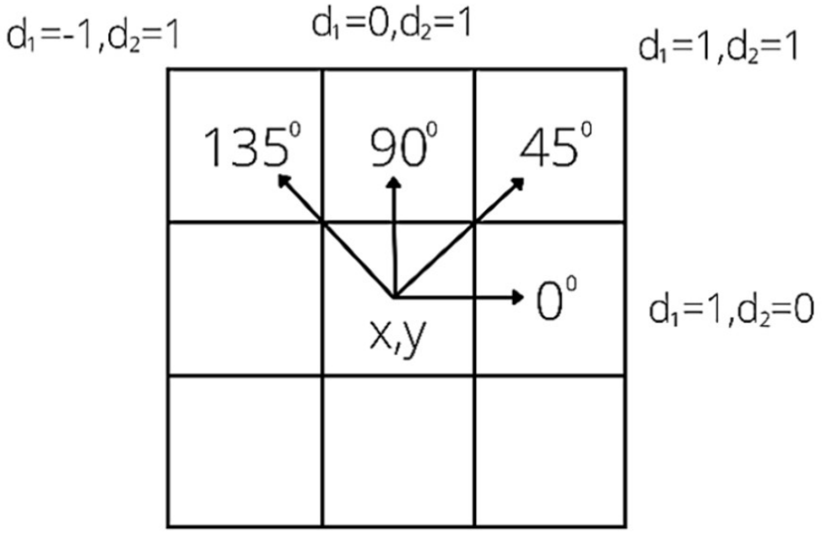


**Supplementary Figure S7.** Coordinate frame with the direction of analysis.

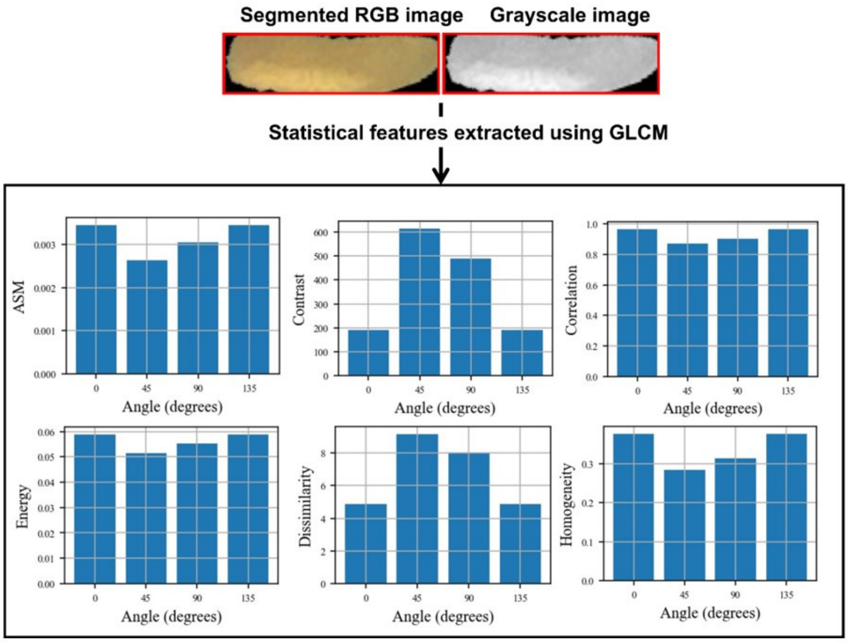


**Supplementary Figure S8.** Statistical features (ASM, Contrast, Correlation, Energy, Dissimilarity, and Homogeneity) extracted using the GLCM for a representative segmented rice grain image.

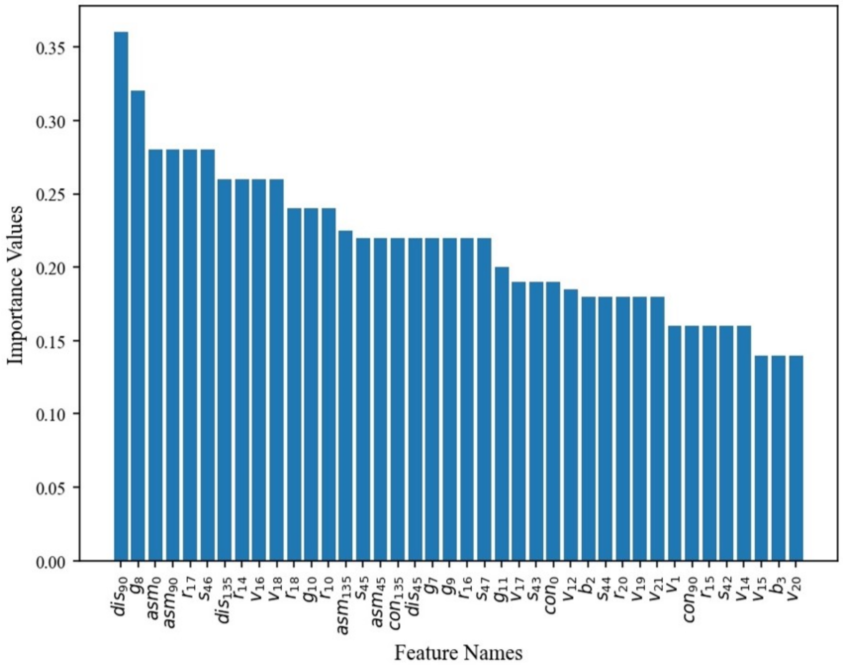


**Supplementary Figure S9.** Key features and their magnitude in terms of their importance value.

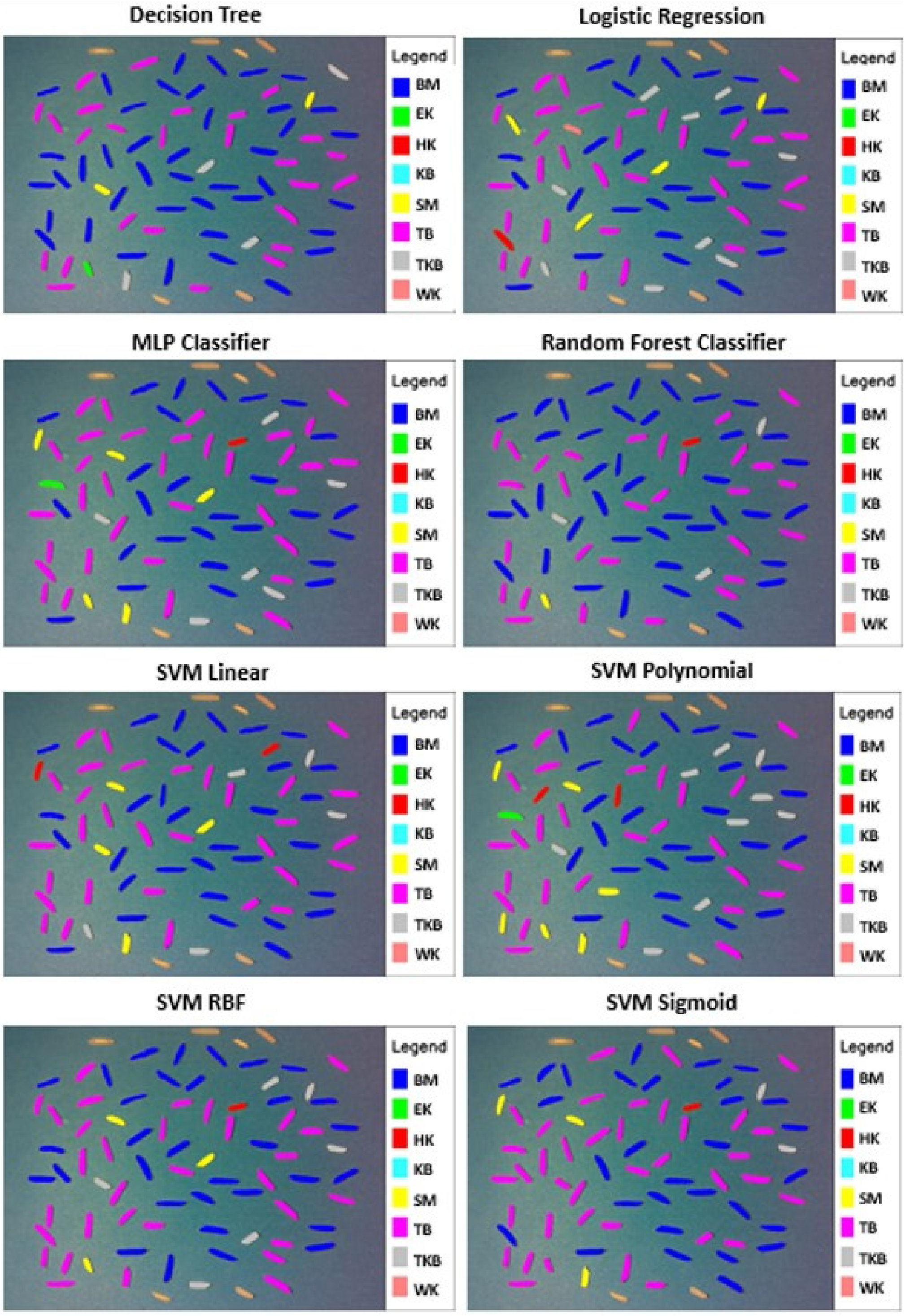


**Supplementary Figure S10.** Labelled rice grain images with different machine learning models, which shows a qualitative comparison between the models.
